# Diagnostic value of cavernous sinus swelling and extrusion sign in cavernous sinus hemangioma

**DOI:** 10.1016/j.heliyon.2024.e26201

**Published:** 2024-02-18

**Authors:** Guoqing Han, Zhifa Huang, Huanhuan Qiao, Wei Zhu, Xuejiang Yan, Ke Pu, Qingguo Li, Xiaoguang Tong

**Affiliations:** a Departments of Neurosurgery, Tianjin University Huanhu Hospital, Tianjin, China; b Departments of Neurosurgery, Tianjin Huanhu Hospital, Tianjin, China; c Tianjin Key Laboratory of Brain Science and Neural Engineering, Academy of Medical Engineering and Translational Medicine, Tianjin University, Tianjin, China

**Keywords:** Cavernous sinus swelling and extrusion sign, Cavernous sinus hemangioma, Light bulb sign, MRI, Gamma Knife

## Abstract

Background and purpose: To examine the diagnostic value of imaging features in cavernous sinus hemangioma (CSH). Materials and Methods: The clinical and imaging data of patients with pathologically confirmed CSH, cavernous sinus meningioma, trigeminal schwannoma and pituitary adenoma invading the cavernous sinus between May 2017 and May 2022 were retrospectively analyzed. The cases were divided into the CSH and non-CSH groups to summarize the magnetic resonance imaging (MRI) characteristics of CSH. Univariate χ2 analysis was performed to assess five indexes, including signal intensity on T2WI, homogeneity of T2WI, enhancement of enhanced T1, enhanced T1 with dural tail sign, and cavernous sinus swelling and extrusion sign. Results: There were significant differences in four features, including hyperintensity on T2WI, homogeneity of T2WI, T1-enhanced without meningeal tail sign, and cavernous sinus swelling and extrusion sign between the CSH and non-CSH groups, with cavernous sinus swelling and extrusion sign showing the most pronounced distinction, with a sensitivity of 100%, a specificity of 93.02%, and an accuracy of 94.23%. The four features could be jointly used as diagnostic criteria, with a sensitivity of 94.44%, a specificity of 100.00%, and an accuracy of 99.04%. Conclusion: Cavernous sinus swelling and extrusion sign is a reliable imaging index for CSH diagnosis. Homogenous hyperintensity or marked hyperintensity on T2WI, enhanced T1 without dural tail sign, and cavernous sinus swelling and extrusion sign could be jointly used as diagnostic criteria, which may improve the accuracy of CSH diagnosis.

## 1 Introduction

Cavernous sinus hemangioma (CSH) is the commonest extracerebral cavernous hemangioma [1]. However, CSH is a rare benign mass, accounting for only 2–3% of benign tumors or tumor-like lesions in the cavernous sinus area [2]; it is often misdiagnosed as meningioma, schwannoma, and invasive pituitary tumor and treated surgically. Currently, total resection rate for CSH microsurgery is relatively low, with heavy bleeding occurring during surgery, elevated disability rate, various complications and certain mortality. Gamma Knife, the preferred treatment option for CSH, safely and effectively controls CSH development and significantly relieves symptoms. Accurate diagnosis of CSH preoperatively to perform Gamma Knife could avoid the serious complications and risks associated with the misdiagnosis of other tumors. Thus, accurate CSH diagnosis preoperatively is of vital importance. In this study, the MRI features of 104 cases with cavernous sinus lesions were retrospectively examined, to improve the accuracy of preoperative diagnosis of CSH.

## 2 Material and methods

The clinical, imaging and surgical data of 104 patients administered cavernous sinus lesion resection in the Department of Neurosurgery of Tianjin Huanhu Hospital from May 2017 to May 2022 were retrospectively analyzed. Inclusion criteria were: (1) a lesion invading the cavernous sinus; (2) pathological diagnosis of CSH, meningioma, schwannoma or pituitary adenoma; (3) complete brain MRI scan and MRI enhancement data. Exclusion criteria were: (1) no lesion invading the cavernous sinus; (2) no pathological diagnosis of CSH, meningioma, schwannoma or pituitary adenoma; (3) incomplete brain MRI data.

GE450w with 3.0T was utilized for preoperative and postoperative imaging. The conventional sequence for head MRI scanning was performed: plain scanning of conventional axial plane, sagittal plane and coronal T1WI and T2WI sequences, and sagittal plane T1WI sequence; enhanced scanning on the axial, sagittal and coronal planes. Gd—DTPA, a paramagnetic contrast agent, was used at 0.15 mmol/kg. The MRI parameters were showed as follows: Axial position of T2WI: Slice Thickness: 5.0 mm, FOV read: 240 mm, FOV phase: 80.0%: NEX: 1, TR:4060 ms, TE: 93.0 ms. Enhanced T1 axis: Slice Thickness: 5.0 mm, FOV read: 230 mm, FOV phase: 100.0%, NEX:1, TR:300 ms, TE: 2.46 ms. By a review of the literature, a list of potentially related imaging features was made, including signal intensity on T2WI, homogeneity of T2WI (defined as no significant signal difference within the tumor or the visible signal difference less than 10% of the tumor”), enhancement of enhanced T1, enhanced T1 with dural tail sign, and cavernous sinus swelling and extrusion sign. The tables were designed. The data were extracted and recorded separately by two neuroimaging experts with more than 20 years of work experience blinded to the pathological diagnosis. In case of disagreement, both discussed to reach a consensus.

Signal intensity on T2WI：According to Elster scoring criteria, 4 and 5 points were defined as mild and marked hyperintensities, respectively (Table 1).

**Table 1:** Elster scoring criteria on signal intensity on T2WI.

Item	Score	T2WI
Hypointense signal	1	Markedly hypointense to gray matter, nearly as dark as cortical bone
	2	Markedly hypointense to cortical gray matter
Isointense signal	3	Nearly isointense with cortical gray matter
	4	Mildly hyperintense to gray matter
Hyperintense signal	5	Markedly hyperintense to gray matter, nearly as bright as the cerebrospinal fluid

Cavernous sinus swelling and extrusion sign was defined as the anterior boundary filling the superior orbital fissure in axial images, with the posterior boundary not exceeding the Michael cavity.

Statistical analysis: IBM SPSS Statistics 26.0 was used in this study. All cases were divided into CSH group and non-CSH group. The chi-square test was performed to compare categorical variables between the two independent samples. P < 0.05 was considered statistically significant.

## 3 Results

Totally 104 cases of cavernous sinus lesions were enrolled from May 2017 to May 2022, all of whom underwent craniotomy microsurgery or endoscopic transnasal tumorectomy. In terms of pathological results (Table 2), there were 18 and 86 cases in the CSH and non-CSH groups, respectively. The non-CSH group included 22 cases of cavernous sinus meningioma, 6 of trigeminal schwannoma and 58 of pituitary adenoma invading the cavernous sinus. The commonest type in the CSH group was Type B CSH. In the non-CSH group, the commonest cavernous sinus meningiomas and pituitary adenomas invading the cavernous sinus were meningothelial meningiomas and gonadotroph cell adenomas, respectively.

**Table 2:** Pathological types of cavernous sinus lesions in 104 cases.

Group	Specific pathological subtypes	n	Gender (numbers of Male: Female)	Mean Age (range) (years)
CSH		18	6:12	58 (42–74)
	Type A (spongy type)	6		
	Type B (mulberry type)	12		
Non-CSH		86	43:43	56 (28–81)
Cavernous sinus meningioma		22	5:17	55 (29–76)
	Meningothelial	12		
	Transitional	3		
	Chordoid	2		
	Fibrous	1		
	Psammomatous	1		
	Anaplastic	1		
	Atypia	1		
	Angiomatous	1		
Trigeminal schwannoma		6	2:4	58 (29–80)
Pituitary adenomas invading the cavernous sinus		58	36:22	56 (28–81)
	Gonadotroph cell adenoma	25		
	ACTH cell adenoma	18		
	Prolactin cell adenoma	7		
	Pituitary null cell adenoma	4		
	Plurihormonal cell adenoma	2		
	GH cell adenoma	2		

From the perspective of demographic features, there were 6 males and 12 females in the CSH group, aged from 42 to 74 years with a mean age of 57.56 years. There were 22 cases (male to female ratio of 5:19) with cavernous sinus meningioma, aged 29–76 years with a mean age of 55.23 years. There were 6 cases (male to female ratio of 1:2) with trigeminal schwannoma, aged 29–80 years with a mean age of 57.5 years. There were 58 cases (male to female ratio of 18:11) with pituitary adenoma invading cavernous sinus, aging from 28 to 81 with mean age of 56.44 years.

Univariate χ2 analysis of the five MRI features showed that there were significant differences in four features, including hyperintensity on T2WI, homogeneity of T2WI, T1-enhanced without meningeal tail sign, and cavernous sinus swelling and extrusion sign, between the CSH and non-CSH groups (Table 3). Cavernous sinus swelling and extrusion sign can be used alone as a diagnostic tool, with a sensitivity of 100%, a specificity of 93.02%, and an accuracy of 94.23%. Marked hyperintensity on T2WI, homogenous hyperintensity or marked hyperintensity on T2WI, enhanced T1 without dural tail sign, and cavernous sinus swelling and extrusion sign could be jointly used as diagnostic criteria, with a sensitivity of 50.00%, a specificity of 100.00%, and an accuracy of 91.35%. Homogenous hyperintensity or marked hyperintensity on T2WI, enhanced T1 without dural tail sign, and cavernous sinus swelling and extrusion sign could be jointly used as diagnostic criteria, with a sensitivity of 94.44%, a specificity of 100.00%, and an accuracy of 99.04% (Table 4).

**Table 3:** Univariate and multivariate analyses of MRI features between the CSH and non-CSH groups.

MRI features	CSH group（n = 18）	Non-CSH group（n = 86）	χ^2^ value	P value
①Marked hyperintensity on T2WI	9	1	–	0.000
②Homogenous hyperintensity on T2WI or marked hyperintensity on T2WI	17	10	–	0.000
③Cavernous sinus swelling and extrusion sign	18	6	–	0.000
④Enhanced T1 without dural tail sign	18	64	–	0.011
⑤Homogenous enhanced T1	6	28	0.004	0.949
①+②+③+④	9	0		0.000
②+③+④	17	0		0.000

**Table 4:** Univariate and multivariate analyses of sensitivity, specificity, positive predictive value, and negative predictive value for CSH detection.

MRI features	Sensibility (%)	Specificity (%)	Positive predictive value (%)	Negative predictive value (%)	Accuracy（%）
①Marked hyperintensity on T2WI	50.00	98.84	90.00	90.43	90.38
②Homogenous hyperintensity on T2WI or marked hyperintensity on T2WI	94.44	88.37	62.96	98.70	89.42
③Cavernous sinus swelling and extrusion sign	100.00	93.02	75.00	100.00	94.23
④Enhanced T1 without dural tail sign	100.00	23.26	21.95	100.00	38.46
①+②+③+④	50.00	100.00	100.00	90.53	91.35
②+③+④	94.44	100.00	100.00	98.85	99.04

## 4 Discussion

CSH has a low incidence, accounting for 2%–3% of benign tumors or tumor-like lesions in the cavernous sinus region [[2], [3], [4]] and 0.4%–2% of all intracranial cavernous hemangiomas [5]. According to previous studies, age at CSH onset is 40–60 years, and the incidence of CSH is higher in women than in men. In the CSH group, there were 6 males and 12 females, aged 42–74 years, with a mean age of 57.56 years, which was corroborated previous studies. Both CSH and cavernous vascular malformations (CMs) consist of blood sinuses surrounded by a single layer of endothelial cells, but their biological features are completely different as well as their treatment options. CM is characterized by lesion enlargement through hemorrhage-absorption-re-bleeding, as a vascular malformation with high endothelial proliferation, migration and tubular activity that is generally removed by craniotomy. However, CSH rarely causes bleeding and generally develops slowly. CHS is currently more likely to be considered a benign neoplastic lesion, and treatment plans encompass surgery and gamma knife.

Goel et al. retrospectively analyzed 45 CSH cases administered surgery from 1992 to 2020, including 39 cases who were treated by the complete epidural approach. The results showed there were 3 deaths due to excessive blood loss and 36 cases with worsening or no recovery from oculomotor dysfunction. With a mean follow-up of 110 months, 3 cases relapsed [6]. Of the 18 CSH cases in this study, 6 had intraoperative blood transfusion above 800 ml with the largest volume of 2800 ml, and no deaths occurred. After the operation, 3 cases had worsened ocular movement dysfunction. Besides, ocular movement dysfunction in the others was not relieved. In conclusion, surgical treatment of CSH has a high risk and causes massive bleeding, with low response rate to symptoms.

Compared with elevated disability rate and certain mortality in craniotomy, radiation therapy has overt advantages in the management of CSH. Pan Li retrospectively analyzed the follow-up data of 53 CHS cases treated with Gamma Knife with an average tumor volume of 13.2 cm³. The peripheral dose of Gamma Knife averaged 13.3Gy. The average durations of imaging and clinical follow-ups were 24 months and 34 months, respectively, and the response rate of the tumors was 100%. After 6 months of GKS treatment, the tumors shrank significantly, and their average volume decreased by 60.2%. The final follow-up volume decreased by averagely 79.5%. In 33 cases the symptoms disappeared or were improved, while 2 cases showed worsened symptoms [7]. Xin et al. retrospectively analyzed the follow-up data of 54 cases with large (20 cm^3^< tumor volume ≤40 cm^3^, diameter of 3–4 cm) and huge (tumor volume >40 cm^3^, diameter >4 cm) CHS [8]. The radiotherapy dose of the target margin was 50 Gy divided into 25 doses. All patients had tumor shrinkage within 3 months of radiotherapy, with an average tumor reduction of 79.7% (range, 48.4–98.5%). There were no patients with tumor progression or recurrence. All cases had symptom improvement within 1–12 months after radiotherapy. In the whole follow-up period, no patients developed any form of permanent complications or symptomatic radiotoxicity.

In conclusion, radiation therapy could be considered the preferred treatment option for small, large and huge CSH cases. However, accurate preoperative diagnosis is the prerequisite for optimal treatment. Due to low incidence, the rate of CHS misdiagnosis is as high as 38.9%. To improve the diagnostic accuracy of CSH and avoid misdiagnosis and associated surgical risk, studies have proposed different views. He Kangmin et al. revealed significant differences in marked hyperintensity on T2WI, homogeneity, dumbbell-like appearance and sellar infiltration between the CSH and non-CSH groups [9]. Francisca Montoya et al. suggested that the combination of hypointensity on T1WI, T2WI and hyperintensity on FLAIR, no dispersion limitation, and homogenous enhancement should place CSH at the top of the differential diagnostic list, especially in case of a "fill" mode in dynamic or delayed imaging [10]. Using a literature review, we generated a list of potentially relevant image features, including signal intensity on T2WI, homogeneity of T2WI, enhancement of enhanced T1, enhanced T1 with dural tail sign, and cavernous sinus swelling and extrusion sign. As shown above, there were significant differences in four features, including hyperintensity on T2WI, homogeneity of T2WI, T1-enhanced without meningeal tail sign, and cavernous sinus swelling and extrusion sign between the CSH and non-CSH groups, of which cavernous sinus swelling and extrusion sign was the most distinctive feature with a sensitivity of 100%, a specificity of 93.02%, and an accuracy of 94.23%. Homogenous hyperintensity or marked hyperintensity on T2WI, enhanced T1 without dural tail sign, and cavernous sinus swelling and extrusion sign could be jointly used as diagnostic criteria, with a sensitivity of 94.44%, a specificity of 100.00%, and an accuracy of 99.04%. Thus, it is believed that CSH could be directly diagnosed using these three features without surgical pathology for direct radiotherapy to avoid craniotomy.

To the best of our knowledge, a role for cavernous sinus swelling and extrusion sign in the diagnosis of CSH has not been reported to date, which was defined as the anterior boundary filling the superior orbital fissure with the posterior boundary not exceeding the Michael cavity. Because CSH originates in the cavernous sinus, it grows like a balloon-like expansion, which is more likely to squeeze into the weak area of the cavernous sinus (e.g., supraorbital fissure) to fill the supraorbital fissure. Besides, the posterior boundary is limited by the posterior wall of the cavernous sinus, preventing CSH from crossing the Michael's cavity into the posterior cranial fossa. Thus, regardless of CSH size, the tumors never cross the dural junction of the cavernous sinuses [6]. In the present study, except for 6 patients with invasive pituitary tumors showing the cavernous sinus swelling and extrusion sign, the non-CSH group did not show this sign.

Totally 100% of CSH cases in He Kangmin et al.’s study had marked hyperintensity on T2WI, which is considered the most distinctive feature of CSH, but only 50.00% of CSH cases in the current study had marked hyperintensity on T2WI. Marked hyperintensity on T2WI had low sensitivity, which is inconsistent with a study by He Kangmin et al. This may be explained by differences in the definition of marked hyperintensity, which indicated a case with 5 points, nearly as bright as the cerebrospinal fluid, according to the Elster scoring criteria in this study. The T2WI signal was similar to that of the cerebrospinal fluid.

**Fig. 1:**
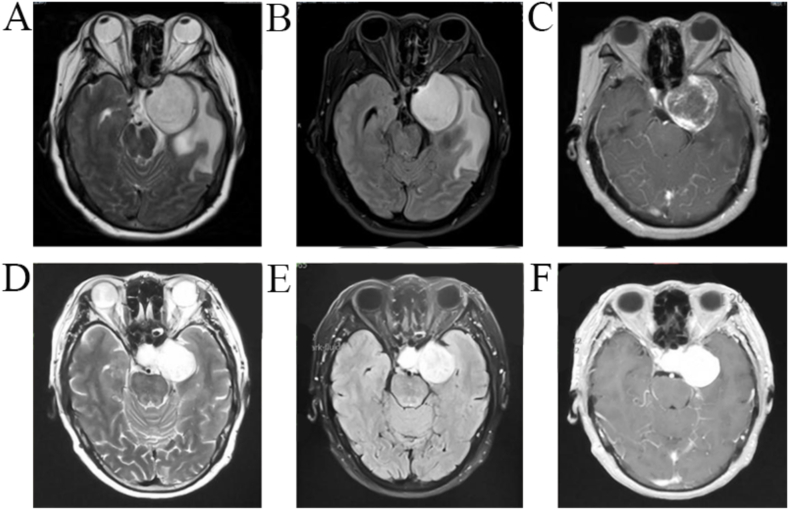
Imaging characteristics of Type A (A, B and C) and Type B (mulberry type) (D, E and F) CSH, revealing significant cavernous sinus swelling and extrusion sign without dural tail sign. (A) Homogenous hyperintensity on T2WI. (B) Hyperintensity on FLAIR. (C) Heterogenous enhancement of enhanced T1 (D) Homogenous marked hyperintensity on T2WI close to the cerebrospinal fluid's signal. (E) Homogenous hyperintensity on FLAIR. (F) Homogenous enhancement of enhanced T1.

Francisca Montoya et al. [10] believed that homogenous enhancement of MR is also helpful in the diagnosis of CSH, but 6 of the 18 cases in the CSH group had homogenous enhancement and 12 did not. Univariate analysis revealed no significant difference in homogenous enhancement between the CSH and non-CSH groups. CSH can be classified as spongy (Type A) and mulberry (Type B) according to pathological features [11,12] (Fig. 1). Type A CSH shows rapid filling and homogenous enhancement, while Type B shows early heterogeneous enhancement and progressive enhancement after delay [9]. In the current study, enhancement homogeneity in CSH cases was consistent with the pathological classification, i.e., all 6 and 12 cases with and without homogenous enhancement were Type A and B CSH, respectively. Thus, homogenous enhancement cannot be used as a MR feature for the detection of CSH, whose enhancement homogeneity is directly related to its pathological subtype.

**Fig. 2:**
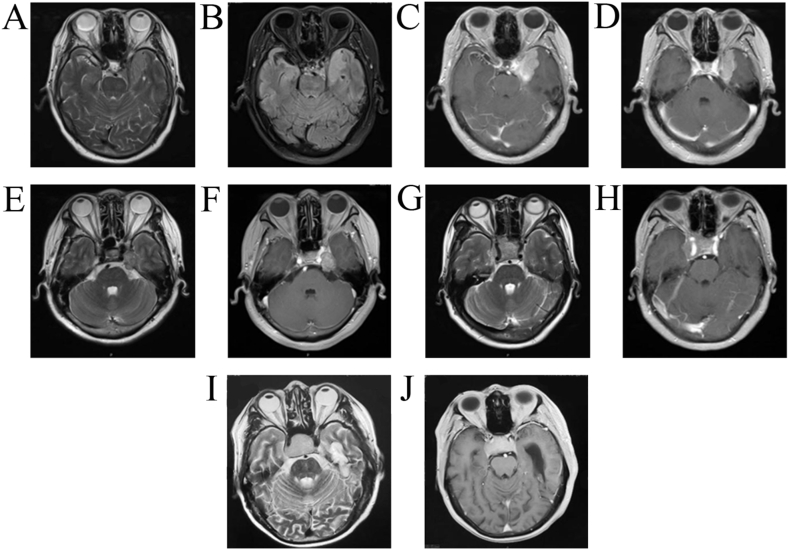
Imaging characteristics of cavernous sinus meningioma (A, B, C, D), schwannoma (E, F) and invasive pituitary adenoma (G, H, I, J). Morphologically, the dural tail sign of cavernous sinus meningioma was obvious with no cavernous sinus swelling and extrusion sign. (A) Heterogenous mild hyperintensity on T2WI. (B) Heterogenous hyperintensity on FLAIR. (C, D) Heterogenous enhancement of enhanced T1. Lesions of schwannoma protrude from the cavernous sinus to the posterior cranial fossa. (E) Heterogenous mild hyperintensity on T2WI. (F) Heterogenous enhancement of enhanced T1 without the dural tail sign of cavernous sinus meningioma or cavernous sinus swelling and extrusion sign. (G, H) Case 1 with heterogenous mild hyperintensity on T2WI and heterogenous enhancement of enhanced T1. (I, J) Case 2 with homogenous mild hyperintensity on T2WI and homogenous enhancement of enhanced T1.

The base of cavernous sinus meningioma, generally originating in the visceral layer of the lateral wall of the cavernous sinus, can invade the cavernous sinus as the tumor grows; however, the tumor's body grows anteriorly and externally along the sphenoid crest, grows posteriorly and externally along the anterior and posterior bedded ligaments, or crosses the cerebellar curtain into the posterior cranial fossa (Fig. 2A, B, 2C and 2D). Unlike CSH, cavernous sinus meningiomas have heterogeneous T2WI with rare marked hyperintensity. Morphologically, cavernous sinus meningiomas generally show the dural tail sign without cavernous sinus swelling and extrusion sign, and sometimes grow posteriorly into the posterior cranial fossa.

Schwannomas are located between the visceral and parietal layers of the lateral wall of the cavernous sinus, generally originating from the semilunar segment of the trigeminal nerve. The tumors grow forward but cannot invade the supraorbital fissure (Fig. 2E and F). In addition, these tumors grow backward and can enter the posterior cranial fossa via the Meckel's cavity. Thus, schwannomas show no cavernous sinus swelling and extrusion sign. Meanwhile, schwannomas rarely show homogenous and marked hyperintensity on T2WI, which easily distinguishes them from CSH.

Pituitary adenomas invading the cavernous sinus generally grow by intrasellar to cavernous sinuses (Fig. 2G, H, 2I and 2J). A few cases of pituitary adenomas invading the cavernous sinus may have cavernous sinus swelling and extrusion sign. In this study, all 6 cases of pituitary adenoma invading the cavernous sinus in the non-CSH group had cavernous sinus swelling and extrusion sign, and all were pituitary adenomas invading the cavernous sinus. However, pituitary adenomas rarely show homogenous and marked hyperintensity on T2WI, which easily distinguishes them from CSH.

## 5 Conclusion

The cavernous sinus swelling and extrusion sign is a reliable marker for the diagnosis of CSH, which has not been reported in previous studies. Homogenous hyperintensity or marked hyperintensity on T2WI, enhanced T1 without dural tail sign, and cavernous sinus swelling and extrusion sign could be jointly used as diagnostic criteria, which may help improve the accuracy of CSH diagnosis.

## Limitations

The research was retrospective and single-center, which there may be confounding and selection bias, so further prospective and multicenter data were needed to test the validity of our prediction model.

## Data availability

Data included in article/supp. material/referenced in article.

## Ethics approval

Not applicable.

## Consent for publication

Not applicable.

## Funding

This work was supported by 10.13039/501100010041Tianjin Science and Technology Planning Project (21JCZDJC00460).

## CRediT authorship contribution statement

**Guoqing Han:** Writing – review & editing, Writing – original draft, Formal analysis, Conceptualization. **Zhifa Huang:** Writing – original draft, Formal analysis, Data curation, Conceptualization. **Huanhuan Qiao:** Software, Methodology. **Wei Zhu:** Validation, Supervision. **Xuejiang Yan:** Validation, Supervision. **Ke Pu:** Visualization, Investigation, Funding acquisition. **Qingguo Li:** Writing – review & editing, Supervision, Project administration, Funding acquisition. **Xiaoguang Tong:** Validation, Supervision.

## Declaration of competing interest

The authors declare that they have no known competing financial interests or personal relationships that could have appeared to influence the work reported in this paper.
